# Solanine Inhibits Immune Escape Mediated by Hepatoma Treg Cells via the TGF*β*/Smad Signaling Pathway

**DOI:** 10.1155/2020/9749631

**Published:** 2020-11-02

**Authors:** Juwei Gao, Yinyin Ying, Jue Wang, Yiyi Cui

**Affiliations:** Department of Oncology, The Third Affiliated Hospital of Zhejiang Chinese Medical University, Hangzhou, 310012 Zhejiang, China

## Abstract

**Objective:**

To observe the inhibitory effect of solanine on regulatory T cells (Treg) in transplanted hepatoma mice and to study the mechanism of solanine inhibiting tumor growth.

**Methods:**

The levels of Treg cells and IL-2, IL-10, and TGF*β* in the blood of patients with liver cancer were detected by flow cytometry and ELISA, respectively. A mouse hepatocellular carcinoma (HCC) graft model was established and randomly divided into four groups: control group, solanine group, TGF*β* inhibitor group (SB-431542), and solanine ^+^TGF*β* inhibitor combined group. Tumor volume of each group was recorded, tumor inhibition rate was calculated, and tumor metastasis was counted. The proportion of CD4^+^CD25^+^Foxp3^+^ Treg in transplanted tumor tissues was detected by flow cytometry. The expression levels of Foxp3 and TGF*β* in transplanted tumor tissues were detected by quantitative fluorescence PCR.

**Results:**

Compared with healthy people, Treg cells and IL-2, IL-10, and TGF*β* contents in peripheral blood of liver cancer patients were increased. The results of the transplanted tumor model in mice showed that the tumor volume of the transplanted mice in the solanine group and the TGF*β* inhibitor mice was reduced compared with the control group. The combined group had the smallest tumor volume. The proportion of CD4^+^CD25^+^Foxp3^+^ Treg in the transplanted tumor tissues of mice in the solanine treatment group was significantly lower than that in the control group. The expressions of Foxp3 and TGF*β* in the transplanted tumor tissues of mice in the solanine group were significantly lower than those in the control group.

**Conclusion:**

Solanine may enhance the antitumor immune response by downregulating the proportion of CD4^+^CD25^+^ Treg and the expression of Foxp3 and TGF*β* in tumor tissues.

## 1. Introduction

The mechanism of HCC development and distant metastasis is very complex, among which immune dysfunction is an important factor [1–5]. Various immune mechanisms can target tumor cells to stimulate the body's immune response to tumor cells, including cellular immunotherapy, cytokines, tumor vaccines, and immunocheckpoint inhibitors [6–8].

CD4^+^CD25^+^ Treg cells are usually divided into two categories according to the differences in cell development, biological characteristics, and mechanism of action [9–11]. These include naturally occurring regulatory T cells (nTreg) and induced (or acquired) regulatory T cells (iTreg) from the thymus [12]. iTreg cells are induced, immunomodulatory cells. Recent studies have shown that in addition to CD4^+^CD25^+^ Treg cells produced by the thymus, peripheral common CD4^+^CD25^+^ T cells can express Foxp3 in vivo and transform into iTreg cells under the conditions of chronic antigen stimulation and cytokines [13, 14]. iTreg cells are derived from peripheral CD4^+^ T cells under the influence of various cytokines and environmental factors after receiving antigen stimulation [15]. CD4^+^CD25^+^ Treg cells are closely related to immune evasion and tolerance of tumors. A large number of studies have found that CD4^+^CD25^+^ Treg cells can be detected in peripheral blood and tumor-infiltrating lymph nodes of patients with gastric cancer, liver cancer, breast cancer, and other cancers [16]. CD4^+^CD25^+^ Treg cells may play an immunosuppressive role by activating them after recognizing tumor antigens and inhibit the production of antitumor immune responses in the body [9–11], to leave the body in a state of low or no response to the tumor. Foxp3, a member of the forkhead transcription factor family, is a specific morphological and functional indicator of human CD4^+^CD25^+^ Treg cells. Foxp3 plays an important role in the development, differentiation, maturation, and function maintenance of Treg cells, and is considered to be the most sensitive marker of CD4^+^CD25^+^ Treg cells [17–19]. As a member of the TGF*β* family of growth factors, TGF*β* can regulate the proliferation and differentiation of immune cells and suppress the immune response. TGF*β* is a growth regulator of a variety of human epithelial-derived cells and plays an important role in the occurrence, development, and metastasis of tumors.

Solanine is a weakly basic glycoside [20, 21]. Studies have shown that solanine has a wide range of anticancer effects in liver cancer, breast cancer, liver cancer, pancreatic cancer, colorectal cancer, and other tumors [22]. Solanine can inhibit cell proliferation, induce cell apoptosis, block cell cycle, induce autophagy, enhance chemoradiotherapy, inhibit epithelial-mesenchymal transformation, inhibit tumor metastasis, and inhibit angiogenesis [21, 23]. However, there are few studies on the effect of solanine on tumor immune function.

In this study, the effects of solanine on CD4^+^CD25^+^ Treg cells in tumor microenvironment of transplanted hepatocellular carcinoma mice were observed, to study the antitumor effect of solanine and to explore the possibility of solanine in immunotherapy for tumor.

## 2. Materials and Methods

### 2.1. Collection of Patient Specimens

Fifteen cases of hepatocellular carcinoma were collected from our hospital from December 2018 to April 2020. Preoperative chemotherapy and immunotherapy were not performed to exclude immune-related diseases. The age ranged from 36 to 68, with a median age of 47. Fifteen healthy subjects excluded tumor and immune-related diseases in hospital physical examination were taken as the control group. All patients signed informed consent forms, and the study was reviewed by the ethics committee of our hospital.

### 2.2. Serum Samples Were Detected by ELISA

Cytokines IL-2, IL-6, and IL-10, TGF*β*, and enzyme-linked immunosorbent assay (ELISA) kits were purchased from Wuhan Baodu Company. In the detection of TGF*β*, the reagent was purchased from Jingmei Bioengineering Co., Ltd. and was detected by Denley automatic labeling analyzer in the United States. Serum TGF*β* was detected by ELISA, and the operation was strict according to the instructions.

### 2.3. Cell Culture and Transfection

RPMI1640 cell culture medium, fetal bovine serum, and lymphocyte separation solution were purchased from Gibco, USA. Human hepatocytes and hepatocellular carcinoma cells were purchased from American Type Culture Collection (ATCC, Manassas, VA, USA). H22 cells were inoculated in culture bottles at appropriate concentrations and added to culture medium containing 10% FBS RPMI1640, cultured in an incubator with constant temperature of 37°C, 5% CO_2_, and saturated humidity. Adherent cell growth, passage every 2~3 days once. At the time of administration, human liver cancer cells at exponential growth stage were taken and digested by 0.25% trypsin. Cell suspension with 10% fetal bovine serum was adjusted to a concentration of 3 × 10^5^/mL and inoculated into 6-well plates with 1 mL per well. The 6-well plate was placed in a carbon dioxide incubator at 37°C and 5% CO_2_. After 24 h of culture, solanine was added, and the final concentrations were 0.4, 2, and 10 *μ*mol/L, respectively.

### 2.4. Xenograft Tumor Model

Eighty female nude mice aged 4 to 5 weeks, with SPF grade, were purchased from the Laboratory Animal Center of Peking University and kept in SPF room. Liver cancer cell H22 was diluted with RPMI1640 cell culture medium to a suspension containing 1 × 10^7^ cells per 200 *μ*L and injected subcutaneously into 3 mice. After continued feeding for 1 week, the subcutaneous eminence of mice was visible, indicating the formation of tumors containing liver cancer tumor cells. After the mice were put to death, the subcutaneous skin was cut off in the superclean workbench after sterilization with medical alcohol, and the disinfected scalpel was cut into small pieces. The cells were washed with sterile PBS solution twice. The nude mice were randomly divided into 4 groups: the tumor blocks were inoculated subcutaneously under the right armpit to about 2 cm above the midaxillary line of the mice, and tumor formation in the mice was observed. When the diameter of the subcutaneous cervical tumor block was 5 mm, the tumor-bearing model was successful.

### 2.5. Administration Method and Dose

In the model control group, 0.2 mL/d of normal saline was intraperitoneally injected. In the solanine group, solanine was intraperitoneally injected at a dose of 37.5 mg/kg, 0.2 mL/day. In the combined TGF*β* inhibitor group, solanine was intraperitoneally injected at a dose of 37.5 mg/kg, 0.2 mL per day, and TGF*β* inhibitor SB-431542 was added on the 6th, 7th, and 13th and 14th days, at a dose of 0.2 mL per 10 mg/kg. In the TGF*β* inhibitor group, 0.2 mL/d of normal saline was intraperitoneally injected. TGF*β* inhibitor SB-431542 was added on the 6th, 7th, and 13th and 14th days, and 0.2 mL of 10 mg/kg dose was intraperitoneally injected.

### 2.6. Flow Cytometry to Detect Treg in Peripheral Blood

Reagents were purchased from Jingmei Bioengineering Co., Ltd. and tested using Coulter Epics XL flow cytometer (Beckman Coulter, USA). 40 *μ*L of EDTA-K2 anticoagulated blood and 5 *μ*L of CD4-PE and CD25-FITC antibodies were added. In the same type of control tube, another 40 *μ*L of blood was added to 5 *μ*L of IgG1-PE and 5 *μ*L of IgG2a-FITC, mixed well, and incubated in the dark at room temperature for 20 min. After hemolysis, add 3 mL of PBS buffer, centrifuge at 1800 r/min for 5 min, and discard the supernatant. Then, add 500 *μ*L of PBS buffer solution and check by flow cytometry. The results are expressed as a percentage.

### 2.7. Treg Cells in Mouse Spleen Lymphocytes Were Detected by Flow Cytometry

The mouse spleen was isolated to make cell suspension. After filtration, the cells were transferred to the new centrifuge tube. Centrifuge at 800 × *g* for 5 min and remove the supernatant. The cells were washed with PBS solution 2 times and then centrifuged to remove supernatant. An appropriate amount of cell culture medium was added to adjust the cell density to 1 × 10^7^/mL. The cells were stratified by the treatment of lymphocyte separation solution, and the lymphocyte layer was the cloud layer between the stratified solution and the supernatant. Draw the needle into the new centrifuge tube. After washing with sterile PBS solution for 2 times, the supernatant was centrifuged to obtain the mononuclear cells of spleen tissue. 100 *μ*L of mononuclear cell suspension of spleen tissue was added into the flow tube with PERCP-labeled CD4 antibody and the second tube with PERCP-labeled CD4 and FITC-labeled CD25. After incubation at room temperature in dark for 20 min, 500 *μ*L of intracellular membrane breaker was added for 10 min, and the supernatant was removed by centrifugation. PE-labeled Foxp3 antibody was added, was incubated in the dark for 20 min, was washed with PBS once, and was suspended. The proportion of CD4^+^CD25^+^Foxp3^+^/CD4^+^ (Treg) cells in mouse lymphocytes in each group was measured by upflow cytometry.

### 2.8. Immunofluorescence

The slides were fixed with 4% paraformaldehyde for 15 min, and the slides were soaked with PBS for 3 times, 3 min each. Triton X-100 (PBS) permeable cells at room temperature for 20 min. Normal goat serum was added to the slides and sealed at room temperature for 30 min. Each slide was dripped with enough diluted primary antibody (Foxp3, Abcam, 1 : 100) and placed in a wet box, incubated at 4°C overnight. Add fluorescent secondary antibody. After the absorbent paper was dried and the excess liquid was added to the slide, the diluted fluorescent secondary antibody was added and incubated at 37°C in a wet box for 1 h. PBST was soaked and sliced for 3 times, 3 min each. DAPI was added for incubation in dark for 30 min, and the specimen was stained. The tablets were sealed with a sealing solution containing an antifluorescent quenching agent. Images were observed and collected under a fluorescence microscope (Nikon, Japan).

### 2.9. Immunohistochemical

The IHC EnVision two-step method was used to detect the expression of Ki-67 in tumor tissue. All fresh specimens were fixed with 10% formaldehyde and sampled within 48 h, paraffin-embedded, and consecutively sectioned, with 4 *μ*m thick. Mouse anti-human Ki-67 monoclonal antibody was purchased from Abcam Company. The working fluid concentration was 1 : 100, and PBS was used as the negative control instead of two primary antibodies. EnVision Kit was purchased from Dako. The sodium citrate buffer (pH = 6.0) was used to repair the antigen by microwave. The rest of the steps strictly follow the instructions.

### 2.10. qRT-PCR

The reverse transcription kit and qPCR kit were purchased from Dalian Takara Company. The total RNA of spleen lymphocytes in each group was extracted by the TRIzol method. Total RNA concentration was determined by spectrophotometry. A cDNA template was obtained by using reverse transcription kit to extract 500 ng of total RNA from mouse lymphocytes. qPCR primers for target genes were designed and synthesized by Shanghai Sangon Co., Ltd.: Foxp3 Sense: 5′-CACCTATGCCACCCTTATCCG-3′, Foxp3 Anti-sense: 5′-CATGCGAGTAAACCAATGGTAGA-3′; GAPDH Sense: 5′-AGGTCGGTGAACGGATTTG-3′, GAPDH Anti-Sense: 5′-GGGGTCGTTGATGGCAACA-3′. Procedures were performed as described in SYBR Premix Ex Taq^TM^ II and tested by the U.S. Bio-Rad iQ5 qPCR system. Reaction conditions are as follows: 94°C, 30 s; 58°C, 30 s; and 72°C, 30 s, with a total of 40 cycles. As an internal reference gene, GAPDH was repeated for 3 times, and the mRNA expression of each group was calculated by the 2^-*ΔΔ*CT^ method.

### 2.11. Statistical Analysis

SPSS 20.0 (SPSS Inc., Chicago, IL, USA) statistical software was used for relevant data analysis. All data were expressed as the mean ± standard deviation. Comparisons between the two groups were performed using the *t*-test. Comparisons between groups were performed using one-way ANOVA. *P* < 0.05 or *P* < 0.01 means that the difference was statistically significant.

## 3. Results

### 3.1. Treg Cells Increased in Peripheral Blood of Tumor Patients, and the Levels of IL-2, IL-10, and TGF*β* Increased

**Figure 1:**
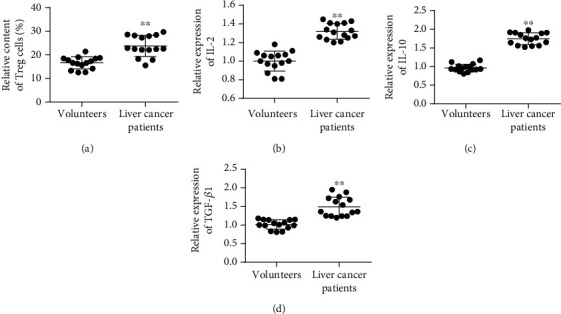
Detection of Treg cell content and TGF*β* in peripheral blood of tumor patients. (a) Treg cell content in serum. (b) IL-2 content in serum. (c) IL-10 content in serum. (d) TGF*β* content in serum. ^∗^*P* < 0.05 and ^∗∗^*P* < 0.01.

In order to study the content of immune cells and the changes of tumor immune-related factors in peripheral blood of patients with liver cancer, we collected the peripheral blood of 15 healthy volunteers and 15 patients with liver cancer. The experimental results showed that compared with healthy volunteers, the proportion of Treg in peripheral blood of liver cancer patients in CD4^+^ T cells was analyzed and cell surface markers were detected on flow cytometry, and the number of Treg in the liver cancer group was higher than that in the control group (*P* < 0.01, Figure 1(a)). Further, ELISA was used to detect the levels of IL-2, IL-10, and TGF*β* in serum. The results showed that the levels of IL-2, IL-10, and TGF*β* in the serum of liver cancer patients were significantly increased compared with healthy volunteers (*P* < 0.01, Figures 1(b)–1(d)).

### 3.2. Liver Cancer Cells Release More TGF*β* than Normal Stem Cells

**Figure 2:**
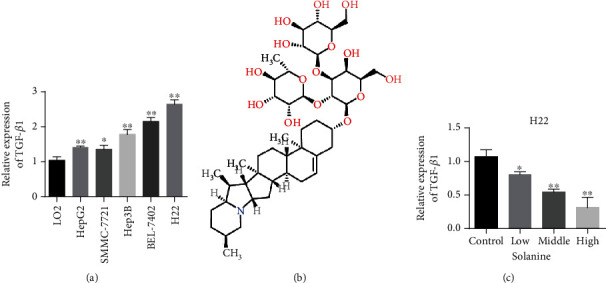
Liver cancer cells release more TGF*β* than normal stem cells. (a) Compared with normal liver cells (LO2) and liver cancer cells (HepG2, SMMC-7721, Hep3B, BEL-7402, and H22), TGF*β* content was detected. (b) Structural formula of solanine. (c) Solanine treats liver cancer cells and inhibits TGF*β*. ^∗^*P* < 0.05 and ^∗∗^*P* < 0.01.

Furthermore, the expression of TGF*β* in liver cancer cell lines and normal liver cells was determined. The experimental results showed that compared with normal liver cells, TGF*β* expression was higher in HCC cell lines, with H22 having the highest TGF*β* expression (Figure 2(a)). In the previous experimental results, we confirmed the inhibitory effect of solanine on liver cancer. In this study, we examined the inhibitory effect of solanine on TGF*β* of hepatocellular carcinoma cell line H22. The results showed that solanine could significantly reduce the content of TGF*β* in H22 with a dose-dependent relationship (Figures 2(b) and 2(c)).

### 3.3. Solanine Inhibits the Growth and Metastasis of Transplanted Hepatoma in Mice

**Figure 3:**
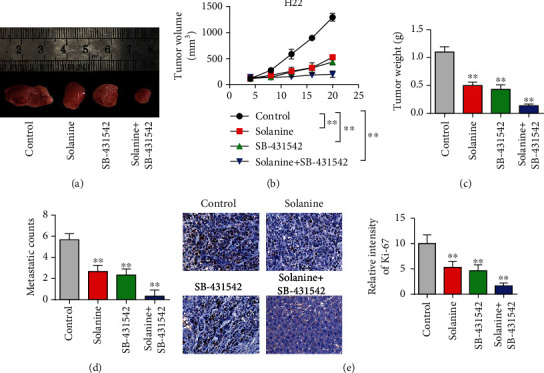
Animal level study of solanine antitumor. (a) Pictures of tumors in a mouse subcutaneous tumor-bearing model. (b) Mouse tumor volume curve. (c) Tumor weight detection in mice. (d) Detection of liver metastasis in mice. (e) Ki-67 immunohistochemical staining of mouse tumor tissue. ^∗^*P* < 0.05 and ^∗∗^*P* < 0.01.

After inoculation of liver cancer cells in mice for 5-7 days, subcutaneous tumor formation could be felt, and tumor lesions could be observed by naked eye on the 7th day or so. The transplanted tumors in the PBS group grew rapidly, and the growth curve was steep (Figures 3(a) and 3(b)). There was no significant difference in the growth rate of transplanted tumors in the solanine group, the TGF*β* inhibitor group, and the combination group compared with the control group at the initial stage of treatment. With the extension of treatment time, the growth of transplanted tumors in mice in the solanine group, the TGF*β* inhibitor group, and the combination group was slow. The tumor weight test results showed that the transplanted weight of mice in the solanine group was significantly lower than that in the control group (Figure 3(c)). The tumor metastasis detection results showed that compared with the control group, tumor metastasis was significantly reduced in the solanine group, the TGF*β* inhibitor group, and the combination group (Figure 3(d)). Further, we detected the cell proliferation index Ki-67 staining by immunohistochemistry. The experimental results showed that compared with the control group, Ki-67 staining of tumor cells was significantly decreased in the solanine group, the TGF*β* inhibitor group, and the combination group, indicating that the solanine group, the TGF*β* inhibitor group, and the combination group could significantly reduce the proliferation ability of tumor cells (Figure 3(e)).

### 3.4. Solanine Treatment Reduced the Proportion of CD4^+^CD25^+^Foxp3^+^ Treg in Transplanted Tumor Tissues

**Figure 4:**
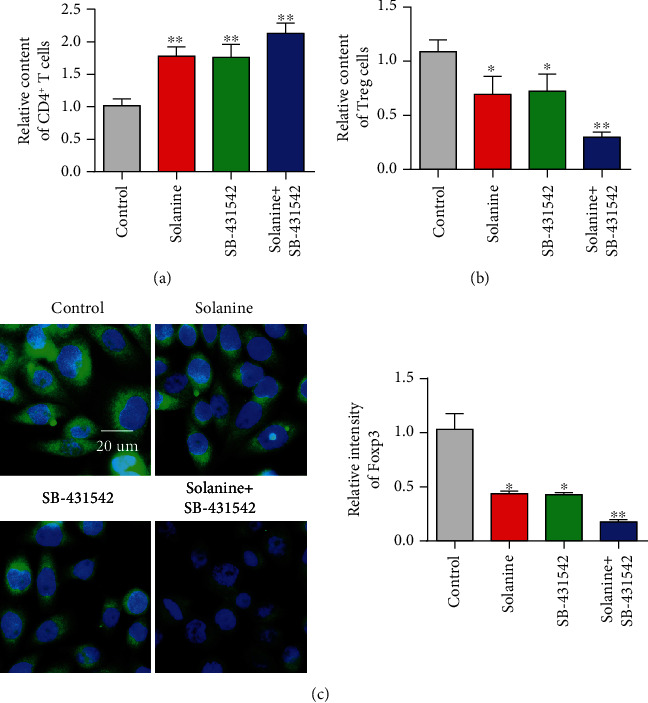
Expression levels of CD4^+^ cells, D4^+^CD25^+^Foxp3^+^ Treg, and Foxp3^+^ immunofluorescence intensity in tumor tissues treated with solanine. (a) Detect the content of CD4^+^ T cells by flow cytometry. (b) Detect the content of CD4^+^CD25^+^Foxp3^+^ Treg by flow cytometry. (c) Foxp3^+^ immunofluorescence intensity of tumor tissue sections. ^∗^*P* < 0.05 and ^∗∗^*P* < 0.01.

**Figure 5:**
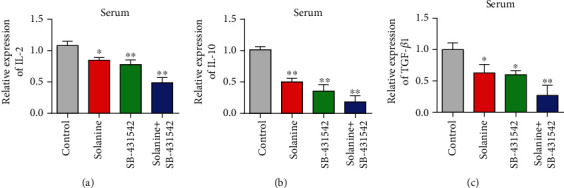
Detection of IL-2, IL-10, and TGF*β* levels in serum of tumor-bearing mice. (a) Detection of IL-2 in serum by qPCR. (b) Detection of IL-10 in serum by qPCR. (c) Detection of TGF*β* in serum by qPCR. ^∗^*P* < 0.05 and ^∗∗^*P* < 0.01.

**Figure 6:**
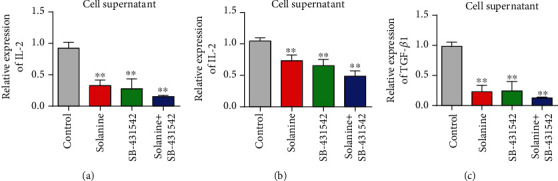
Levels of IL-2, IL-10, and TGF*β* in spleen CD4^+^ cell culture supernatant of tumor-bearing mice. (a) Detection of IL-2 content in the supernatant of CD4^+^ T cells by qPCR. (b) Detection of IL-10 in CD4^+^ T cell supernatant by qPCR. (c) qPCR detection of TGF*β* content in CD4^+^ T cell supernatant. ^∗^*P* < 0.05 and ^∗∗^*P* < 0.01.

In order to observe the effect of solanine on Treg in hepatocellular carcinoma-bearing mice, CD4^+^CD25^+^Foxp3^+^ was used as the marker of Treg in this study. CD4 coil gates were first used; then, CD25 coil gates were used to detect the proportion of CD4^+^CD25^+^Foxp3^+^ Treg in CD4^+^ T cells in each group. The proportion of CD4^+^CD25^+^Foxp3^+^ Treg in CD4^+^ T cells in the solanine group, the TGF*β* inhibitor group, and the combination group was significantly lower than that in the control group (Figures 4(a) and 4(b)). At the same time, immunofluorescence staining results showed that the expression of Foxp3 in Treg cells of the solanine group, the TGF*β* inhibitor group, and the combination group was also significantly decreased (Figure 4(c)). At the same time, we also found that solanine treatment reduced the contents of IL-2, IL-10, and TGF*β* in the serum of tumor-bearing mice (Figures 5(a)–5(c)). The Treg cells in the control group, the solanine group, the TGF*β* inhibitor group, and the combined group were separated by flow cytometry and then cultured to detect the contents of IL-2, IL-10, and TGF*β* in the cell supernatant. The results showed that the levels of IL-2, IL-10, and TGF*β* in Treg cells isolated from the solanine group, the TGF*β* inhibitor group, and the combined treatment group were significantly lower than those in the control group (Figures 6(a)–6(c)).

### 3.5. Solanine Inhibited the Expression of Foxp3 and TGF*β* mRNA in Transplanted Tumor Tissues

**Figure 7:**
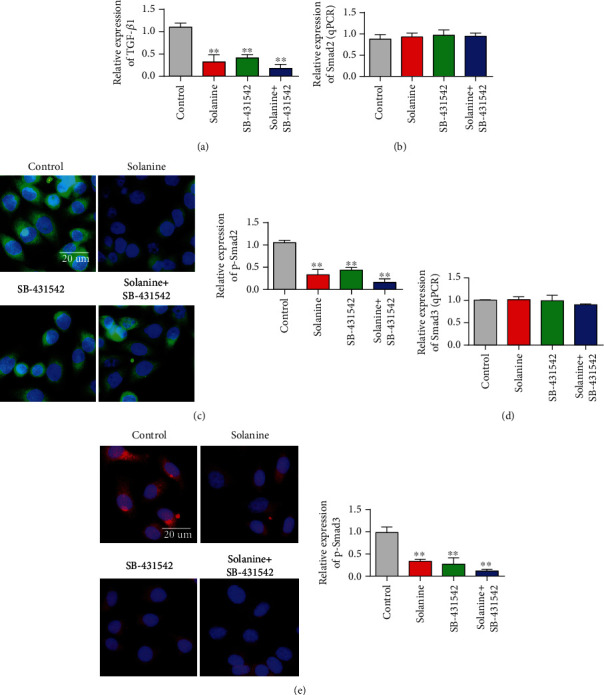
Detected the TGF*β*/Smad3 signaling pathway. (a) Detection of TGF*β* content in sorted cells. (b) Detection of Smad2 content in sorted cells. (c) Detection of p-Smad2 content in sorted cells. (d) Detection of Smad3 content in sorted cells. (e) Detection of p-Smad3 content in sorted cells. ^∗^*P* < 0.05 and ^∗∗^*P* < 0.01.

In order to further investigate the molecular mechanism of solanine in the treatment of liver cancer, we detected the changes of the TGF*β*/Smad signaling pathway in tumor tissues after treatment with solanine. The experimental results showed that compared with the control group, TGF*β* expression was significantly reduced in the solanine group, the TGF*β* inhibitor group, and the combination group, with the maximum reduction in the combination group (Figure 7(a)). Further test results showed that the overall expression of Smad2 did not change significantly, but the p-Smad2 expression decreased significantly (Figures 7(b) and 7(c)). The detection results of Smad3 were consistent with those of Smad2. The overall expression level of Smad2 in the solanine group, the TGF*β* inhibitor group, and the combination group did not change significantly, but the expression level of p-Smad2 was significantly decreased, and the reduction degree of the combination group was the largest (Figures 7(d) and 7(e)).

## 4. Discussion

Hepatocellular carcinoma (HCC) is one of the most common malignancies and ranks the second leading cause of cancer death worldwide [24–26]. Immunotherapy is one of the important means of tumor therapy. In recent years, it has been found that the occurrence, development, and prognosis of tumors are closely related to CD4^+^CD25^+^ Treg cells. Treatment measures against Treg in vivo are obviously beneficial to antitumor immunity [27–30]. Ideal tumor therapeutic effects can be achieved by removing Treg cells in tumor patients and regulating the migration, distribution, and function of Treg. Tumor cells retain the antigenicity of the original cells and produce tumor antigens during carcinogenesis. Therefore, the immune escape of the tumor results in the body's unresponsiveness to the tumor or low immune function. Treg cells can inhibit the development and activation of effector cells that recognize their own tumor cells, and play an important role in mediating tumor immune tolerance. Therefore, inhibition of Treg cells is of great significance for tumor immunotherapy [31–34].

Treg cells are involved in tumor escape by secreting inhibitory cytokines. Treg cells mainly secrete TGF*β* and IL-10 to inhibit the body's antitumor immunity. IL-10 promotes tumor immune escape by inhibiting the function of APC, Th1 cells, NK cells, and macrophages. TGF*β* also induces excessive IL-10 secretion in tumors, leading to immunosuppression of the antitumor response. Chen et al. [35] found that CD4^+^CD25^+^ Treg cells could inhibit the tumor clearance effect mediated by CD8^+^ T cells. TGF*β* plays a key role in this inhibitory process. Treg cells promote tumor immune escape by interacting with immune cells in the tumor microenvironment. Treg cells inhibit tumor immunity by inhibiting tumor-infiltrating T cells (TIL), dendritic cells, and macrophages in the tumor microenvironment. Treg cells inhibit proliferation and induce immune incompetence by competing with effector T cells to bind IL-2 or acting directly on them. IL-2 is a signal of cell proliferation. CD4^+^CD25^+^ Treg cells can upregulate the expression of IL-2 receptor chain, while interfering with the expression of IL-2 receptor chain in effector T cells. Finally, IL-2 receptor can be used competitively, so that effector cells cannot get growth signal and cannot proliferate. In this study, it was found that solanine and TGF*β* inhibitors could significantly inhibit Treg cell content and reduce IL-2 and IL-10 levels.

In this study, a mouse model of hepatocellular carcinoma was successfully established, and it was found that solanine could significantly inhibit the growth of hepatocellular carcinoma in vivo. With the extension of time, the rate of tumor inhibition increased. The results of animal experiments verified the inhibitory effect of solanine on liver cancer. Foxp3 is the most specific marker of Treg cells and plays a key role in the differentiation and function regulation of Treg cells. In this study, qPCR was used to detect gene expression changes of key transcription factors of Treg cells in spleen lymphocytes of hepatoma-bearing mice. The results showed that the expression level of Foxp3 mRNA in lymphocytes of mice in the tumor-bearing model group increased significantly, while the expression level of Foxp3 mRNA in the solanine treatment group decreased significantly, with a time effect [36, 37]. This suggests that solanine may play an immunomodulatory role by inhibiting the expression of Foxp3, a key transcription factor in Treg cells [38, 39]. At the same time, it was also found in this study that the levels of Treg-related immune cytokines (IL-2, IL-10, and TGF*β*) were significantly increased in mice in the hepatoma-bearing model group. However, Treg cell-related immune cytokines decreased after treatment with solanine. This indicated that solanine could reduce Treg cell content in spleen lymphocytes of hepatocellular carcinoma-bearing mice by inhibiting Treg cell-related immune cytokines.

## 5. Conclusion

The results of this study showed that solanine inhibited the growth of transplanted hepatoma in mice and reduced the proportion of CD4^+^CD25^+^Foxp3^+^ Treg and the expression levels of Foxp3 and TGF*β* mRNA. Mechanism studies have shown that solanine reduces the proportion of local CD4^+^CD25^+^Foxp3^+^ Treg in mice and enhances the body's antitumor immune response by inhibiting the TGF*β*/Smad signaling pathway. Therefore, as a potential drug for effective treatment of liver cancer, solanine has potential application prospect. This study provides experimental basis for the future clinical application of solanine.

## Data Availability

All the experimental data can be made public by the requirements of qualified researchers.

## References

[1] Yi C., Yang G., Sun M., Chang K., Long R., Jiang Z. (2017). The Expression and Clinical Significance of Treg Cells in Chronic Myelocytic Leukemia. *Biomedical Research*.

[2] Wu X., Peng M., Huang B. (2013). Immune microenvironment profiles of tumor immune equilibrium and immune escape states of mouse sarcoma. *Cancer Letters*.

[3] Whiteside T. L., Mandapathil M., Szczepanski M., Szajnik M. (2011). Mechanisms of tumor escape from the immune system: adenosine-producing Treg, exosomes and tumor-associated TLRs. *Bulletin du Cancer*.

[4] Meng J., Ai X., Lei Y. (2019). USP5 promotes epithelial-mesenchymal transition by stabilizing SLUG in hepatocellular carcinoma. *Theranostics*.

[5] Meng J., Chen S., Lei Y. Y. (2019). Hsp90*β* promotes aggressive vasculogenic mimicry via epithelial-mesenchymal transition in hepatocellular carcinoma. *Oncogene*.

[6] Shionoya Y., Kanaseki T., Miyamoto S. (2017). Loss of tapasin in human lung and colon cancer cells and escape from tumor-associated antigen-specific CTL recognition. *Oncoimmunology*.

[7] Toor S. M., Elkord E. (2018). Therapeutic prospects of targeting myeloid-derived suppressor cells and immune checkpoints in cancer. *Immunology & Cell Biology*.

[8] Terme M., Pernot S., Marcheteau E. (2012). VEGF-a-induced Treg proliferation, a novel mechanism of tumor immune escape in colorectal cancer: effects of anti-VEGF/VEGFR therapies. *Annals of Oncology*.

[9] Mu C., Zhang G., Huang J., Qi B. (2014). pDC induced Treg proliferation through PD-1/PD-L1 signal and promote tumor immune escape of lung cancer with MPE. *European Respiratory Journal*.

[10] Mocellin S., Nitti D. (2008). Therapeutics targeting tumor immune escape: towards the development of new generation anticancer vaccines. *Medicinal Research Reviews*.

[11] Miyazaki T., Ikeda K., Sato W., Horie-Inoue K., Inoue S. (2018). Extracellular vesicle-mediated EBAG9 transfer from cancer cells to tumor microenvironment promotes immune escape and tumor progression. *Oncogene*.

[12] Bluestone J. A., Abbas A. K. (2003). Natural versus adaptive regulatory T cells. *Nature Reviews Immunology*.

[13] Apostolou I., Von Boehmer H. (2004). In vivo instruction of suppressor commitment in naive T cells. *The Journal of Experimental Medicine*.

[14] Curotto de Lafaille M. A., Lino A. C., Kutchukhidze N., Lafaille J. J. (2004). CD25− T cells generate CD25+ Foxp3+ regulatory T cells by peripheral expansion. *The Journal of Immunology*.

[15] Facciabene A., Motz G. T., Coukos G. (2012). T-regulatory cells: key players in tumor immune escape and angiogenesis. *Cancer Research*.

[16] Wolf A. M., Wolf D., Steurer M., Gastl G., Gunsilius E., Grubeck-Loebenstein B. (2003). Increase of regulatory T cells in the peripheral blood of cancer patients. *Clinical Cancer Research*.

[17] Miyara M., Sakaguchi S. (2011). Human FoxP3+ CD4+ regulatory T cells: their knowns and unknowns. *Immunology & Cell Biology*.

[18] Kleinewietfeld M., Starke M., Di Mitri D. (2009). CD49d provides access to “untouched” human Foxp3+ Treg free of contaminating effector cells. *Blood*.

[19] Lu L., Ma J., Wang X. (2010). Synergistic effect of TGF-beta superfamily members on the induction of Foxp3+ Treg. *European Journal of Immunology*.

[20] Lv C., Kong H., Dong G. (2014). Antitumor efficacy of *α*-solanine against pancreatic cancer in vitro and in vivo. *Plos One*.

[21] Mohsenikia M., Alizadeh A. M., Khodayari S. (2013). The protective and therapeutic effects of alpha-solanine on mice breast cancer. *European Journal of Pharmacology*.

[22] Zhong W. F., Liu S. P., Pan B., Tang Z. F., Zhong J. G., Zhou F. J. (2016). Solanine inhibits prostate cancer Du145 xenograft growth in nude mice by inducing cell cycle arrest in G1/S phase. *Nan Fang Yi Ke Da Xue Xue Bao*.

[23] Zhang J., Shi G. W. (2011). Inhibitory effect of solanine on prostate cancer cell line PC-3 in vitro. *National Journal of Andrology*.

[24] Wang H., Zhong W., Zhao J. (2019). Oleanolic acid inhibits epithelial-mesenchymal transition of hepatocellular carcinoma by promoting iNOS dimerization. *Molecular Cancer Therapeutics*.

[25] Xiao T., Zhong W., Zhao J. (2018). Polyphyllin I suppresses the formation of vasculogenic mimicry via Twist1/VE-cadherin pathway. *Cell Death & Disease*.

[26] Zhong W., Yang W., Qin Y. (2019). 6-Gingerol stabilized the p-VEGFR2/VE-cadherin/*β*-catenin/actin complex promotes microvessel normalization and suppresses tumor progression. *Journal of Experimental & Clinical Cancer Research*.

[27] Hahne M. (1997). Melanoma cells express Fas ligand: implications for tumor immune escape. *Immunology Letters*.

[28] Igney F. H., Krammer P. H. (2002). Immune escape of tumors: apoptosis resistance and tumor counterattack. *Journal of Leukocyte Biology*.

[29] Hahne M., Rimoldi D., Schroter M. (1996). Melanoma cell expression of Fas(Apo-1/CD95) ligand: implications for tumor immune escape. *Science*.

[30] Campoli M., Chang C. C., Ferrone S. (2003). HLA class I antigen loss, tumor immune escape and immune selection. *Vaccine*.

[31] Engel J. R. B., Honig A. (2014). Mechanisms of tumor immune escape in triple-negative breast cancers (TNBC) with and without mutated BRCA 1. *Archives of Gynecology & Obstetrics*.

[32] Buzyn A., Petit F., Ostankovitch M. (1999). Membrane-bound Fas (Apo-1/CD95) ligand on leukemic cells: a mechanism of tumor immune escape in leukemia patients. *Blood*.

[33] Garcia-Lora A., Algarra I., Garrido F. (2003). MHC class I antigens, immune surveillance, and tumor immune escape. *Journal of Cellular Physiology*.

[34] Xi X., Liu N., Wang Q. (2019). ACT001, a novel PAI-1 inhibitor, exerts synergistic effects in combination with cisplatin by inhibiting PI3K/AKT pathway in glioma. *Cell Death & Disease*.

[35] Chen M.-L., Pittet M. J., Gorelik L. (2005). Regulatory T cells suppress tumor-specific CD8 T cell cytotoxicity through TGF-*β* signals in vivo. *Proceedings of the National Academy of Sciences*.

[36] Horwitz D. A., Zheng S. G., Wang J., Gray J. D. (2008). Critical role of IL-2 and TGF-*β* in generation, function and stabilization of Foxp3+CD4+ Treg. *European Journal of Immunology*.

[37] Duhen T., Duhen R., Lanzavecchia A., Sallusto F., Campbell D. J. (2012). Functionally distinct subsets of human FOXP3+ Treg cells that phenotypically mirror effector Th cells. *Blood*.

[38] Palomares O., Rückert B., Jartti T. (2012). Induction and maintenance of allergen-specific FOXP3+ Treg cells in human tonsils as potential first-line organs of oral tolerance. *Journal of Allergy and Clinical Immunology*.

[39] Schneider A., Rieck M., Sanda S., Pihoker C., Greenbaum C., Buckner J. H. (2008). The effector T cells of diabetic subjects are resistant to regulation via CD4+FOXP3+regulatory T cells. *Journal of Immunology*.

